# Loss of STING expression is prognostic in non–small cell lung cancer

**DOI:** 10.1002/jso.26804

**Published:** 2022-01-31

**Authors:** Zoltan Lohinai, David Dora, Charles Caldwell, Christopher J. Rivard, Kenichi Suda, Hui Yu, Gareth Rivalland, Kim Ellison, Leslie Rozeboom, Rafal Dziadziuszko, Paul Mitchell, Thomas John, Inigo S. Millan, Shengxiang Ren, Fred R. Hirsch

**Affiliations:** ^1^ National Korányi Institute of Pulmonology Budapest Hungary; ^2^ Department of Anatomy, Histology, and Embryology, Faculty of Medicine Semmelweis University Budapest Hungary; ^3^ Departments of Medicine and Medical Oncology University of Colorado Anschutz Medical Campus Aurora Colorado USA; ^4^ Division of Thoracic Surgery, Department of Surgery, Faculty of Medicine Kindai University Osaka Japan; ^5^ Olivia Newton‐John Cancer and Wellness Centre, Austin Hospital Heidelberg Victoria Australia; ^6^ Department of Oncology and Radiotherapy Medical University of Gdansk Gdansk Poland; ^7^ Department of Medicine, Metabolism, and Diabetes University of Colorado School of Medicine Aurora CO USA; ^8^ Department of Human Physiology and Nutrition University of Colorado Colorado Springs Colorado USA; ^9^ Department of Medical Oncology, Shanghai Pulmonary Hospital Tongji University School of Medicine Shanghai China; ^10^ Tisch Cancer Institute, Center for Thoracic Oncology Mount Sinai Health System New York New York USA

**Keywords:** cGAS, non–small cell lung cancer (NSCLC), STING, T cell function genes

## Abstract

**Background:**

Stimulator of interferon (IFN) genes (STING) is a protein that promotes type I IFN production essential for T‐cell activation. In this study, we aim to characterize STING expression comprehensively using The Cancer Genome Atlas (TCGA) database, cell lines, and patient tumor samples stained with immunohistochemistry.

**Methods:**

Two cohorts were evaluated comprising 721 non–small cell lung cancer (NSCLC) patients and 55 NSCLC cell lines for STING and cyclic GMP‐AMP synthase (cGAS) expression using immunohistochemistry. Moreover, an independent cohort of *n* = 499 patients from the TCGA database was analyzed. Methylation was evaluated on STING and cGAS in five STING‐negative NSCLC cell lines.

**Results:**

STING RNA expression positively correlates with T cell function and development genes, negatively correlates with cell proliferation and associated with increased survival (5‐year‐overall survival [OS] 47.3% vs. 38.8%, *p* = 0.033). STING protein expression is significantly higher in adenocarcinoma (AC) and is lost with increasing stages of AC. STING‐positivity is significantly higher in mutant EGFR and KRAS tumors. STING‐positive NSCLC patients identified with immunohistochemistry (H‐score > 50) have increased survival (median OS: 58 vs. 35 months, *p* = 0.02). Treatment of STING‐negative cell lines with a demethylating agent restores STING expression.

**Conclusions:**

STING is ubiquitously expressed in NSCLC and associated with T cell function genes, AC histology, EGFR, and KRAS mutations and improved overall survival.

## 1 INTRODUCTION

Controlling anticancer immune response through activating deserted immune tumors to a hot phenotype might enhance the current therapeutic paradigm. The treatment for patients with non–small cell lung cancer (NSCLC) has been changed by introducing anti‐programmed death‐1 (PD‐1) immunotherapy. Long‐term responses in advanced‐stage disease that were previously not anticipated now have been accomplished, and 5‐year overall survival (OS) increased to 20% in unselected and up to 40% in PD‐L1^high^ expressing patients.^1^, ^2^, ^3^, ^4^ Nevertheless, patients now treated with various anti‐PD immunotherapies in the frontline, advanced NSCLC still have poor outcomes, and even with curative resection, 30%–55% of patients with limited‐stage disease relapse.^5^ Thus, new innovative approaches to select patients for therapy that can cost‐effectively be integrated into routine practice that complement current treatments are required. The tumor microenvironment (TME), the interaction of immunotherapy with the balance of the altering immune‐ and therapy response, is a novel direction to enhance treatment efficacy.

STING (Stimulator of Interferon [IFN] Genes, tmem173, MITA, and MYPS) is a protein responsible for controlling anticancer immune responses to leaked self‐ or non‐self DNA.^6^ STING is a transmembrane component of the endoplasmic reticulum that produces type I IFNs (IFNα/β) essential for activating dendritic cells and thus antigen presentation and T‐cell priming.^7^, ^8^, ^9^ Agonists of STING that spike IFN production and show potent immune response are currently under investigation in clinical trials and are of particular interest in combination with checkpoint therapies targeting pathways such as PD‐L1 or cytotoxic T‐lymphocyte‐associated protein 4 (CTLA4).^10^, ^11^ Importantly, recent studies have shown in animal models that knocking out STING and cGAS expression results in a nonresponse to PD‐L1 checkpoint therapy, whereas control mice responded well to PD‐L1 checkpoint inhibition.^12^ STING and cGAS are thus thought to be essential for the antitumor response of PD‐1/PD‐L1 checkpoint inhibition.

Recent studies in hepatocellular,^13^ gastric^14^ and colorectal cancer,^15^, ^16^ and in melanoma^17^ have shown that STING expression was decreased in tumor, compared with healthy tissues. Additionally, STING is frequently lost during tumor progression, and loss of STING/cGAS correlates with poor survival. One common reported mechanism of STING or cGAS loss in tumors is due to upregulated methylation of their respective promoter regions.^10^

The cytoplasmic DNA sensor cGAS (cyclic GMP‐AMP synthase, and MB21D1)^18^ can detect leaked self or non‐self DNA and, in response, will synthesize the cyclic dinucleotide (CDN) cyclic GMP‐AMP.^19^ cGAMP binds STING specifically, activating STING and causing dramatic conformational changes and translocation of STING from the ER to the perinuclear area, where STING acts as an adapter protein essential for immune signaling following the detection of tumor DNA.

Our study aims to analyze the presence and the expression landscape of STING and cGAS protein according to key clinicopathological parameters, including stage, sex, histological type, mutational status, and survival. Furthermore, to validate our results on the human tissues, we analyzed the methylation of STING and cGAS in NSCLC cell lines using demethylating agents.

## 2 MATERIALS AND METHODS

### 2.1 Patient cohorts and tissue microarrays

**Figure 1:**
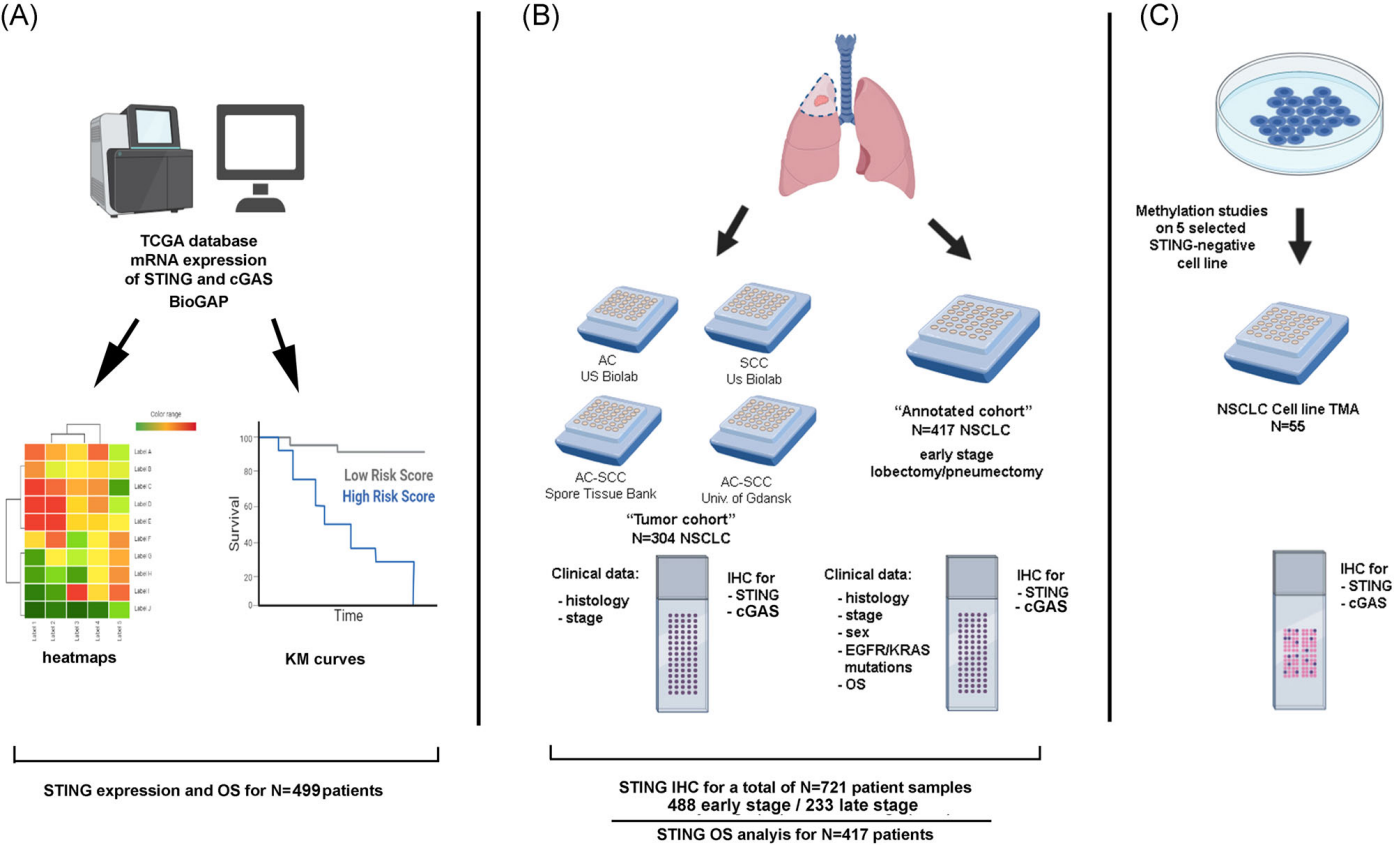
Flowcharts show experimental design, cohorts, and inclusion criteria of the study. Workflow, clinical data availability, and case numbers are presented for TCGA analysis (A), study on the clinical patient cohorts (B), and cell lines (C). cGAS, cyclic GMP‐AMP synthase; IHC, immunohistochemistry; NSCLC, non–small cell lung cancer; STING, stimulator of interferon genes

Tumor tissue samples from a total of 721 patients were included in our study (*N* = 419 AC and *N* = 302 SCC, Table S1) from four sources (Figure 1B). The “tumor cohort” of 304 NSCLC human tumor tissues included clinical data on histology and stage. Within this cohort were two tissue microarrays (TMAs, one adenocarcinoma [AC], and one squamous cell carcinoma [SCC]) purchased from US Biolab and one further TMA (AC and SCC) from the SPORE tissue bank. A third TMA set containing AC and SCC samples with triplicate 1‐mm‐cores was obtained from the Medical University of Gdansk.

The “annotated cohort” including extensive patient data on histology, stage, age, gender, and KRAS and EGFR mutational status was provided by collaborators at Olivia Newton‐John Cancer and Wellness Centre and comprised 417 mostly early‐stage NSCLC cases (*N* = 233 AC, *N* = 141 SCC, and *N* = 43 Other, Table S2) sequentially resected between 1992 and 2010. The included cases underwent lobectomy or pneumonectomy, excluding patients with sublobar resections. Detailed clinicopathologic data were collected prospectively. An experienced thoracic histopathologist selected the areas from blocks to obtain triplicate 1‐mm‐cores for the preparation of TMAs (MP10 1.0 mm tissue punch on a manual TMA instrument; Beecher Instruments). Treatments were administered according to the contemporary NCCN guidelines across all centers included in our study.

### 2.2 Immunohistochemistry (IHC) and scoring

Cell lines, tissues, and TMA sections were stained for immunohistochemistry (IHC) using the Ventana Benchmark XT autostainer, like previously described.^20^ STING antibody (Cell Signaling #13647) was diluted at 1:400 for IHC using Signal Stain antibody diluent (Cell Signaling #8112). cGAS antibody (Novus Biologics #NBP1‐86761) was diluted at 1:300 with Signal Stain diluent. Scoring of STING and cGAS IHC was based on a percentage of tumor cell marker expression (0%–100%) multiplied by staining intensity (0, 1+, 2+ and 3+) to provide an H‐score (range 0–300). H‐scores for multiple cores were averaged. In line with previously reported scoring methods, a cutoff below an H‐score of 50 was considered negative. cGAS expression at ≥1 was identified as positive based on tumor cell staining. Scoring was carried out by two independent observers with software‐assisted (ImageJ) manual cell counting. and specimens were evaluated using an Olympus BX43 brightfield microscope and Olympus DP71 camera and cellSens software.

### 2.3 Cell line studies and immunoblotting

Cell lines used in studies were obtained from ATCC and fingerprinted for authenticity and certified as *Mycoplasma*‐free by the Cell Technologies Shared Resource (CTSR) at the University of Colorado Cancer Center. For methylation studies, all cell lines were grown to 70% confluency in duplicate T25 flasks with Roswell Park Memorial Institute 1640 media containing 10% fetal bovine serum and Pen/Strep. Cell media was then replaced with media alone or media containing 10 µM 5‐Aza‐2ʹ‐deoxycytidine demethylating agent (Sigma‐Aldrich) for 72 h. Cell lines were then lyzed with Cell Lysis Buffer (Cell Signaling, #9803), followed by sonication and centrifugation. Cell lysate protein concentration was determined using the BCA assay (Pierce, #23225).

Western blot analysis was performed using 4%–20% precast Criterion gels (Bio‐Rad, #3450033). Specimens were loaded with 40 µg total protein per lane and run at 70 V for 3 h, followed by protein transfer to polyvinylidene difluoride membrane (Bio‐Rad, #162‐0177) for 500 mAmp*h. Primary antibody was diluted in milk/T‐Tris‐buffered saline (T‐TBS)  and added to membranes, and incubated overnight at 4°C on a rocker, followed by horseradish peroxidase‐secondary antibody after washing with T‐TBS. Membranes were revealed using the Clarity ECL reagent (#1705061, Bio‐Rad) and recorded on radiographic film.

### 2.4 Analysis of The Cancer Genome Atlas (TCGA) database

Provisional LUAD and LUSC data sets from The Cancer Genome Atlas (TCGA) were analyzed for messenger RNA (mRNA) expression of STING using the proprietary program BioGAP. TCGA Methylation data were generated using Illumina 450k Methylation data obtained from cBioPortal (www.cbioportal.org). Heat maps of gene sets were generated using UCSC Xena (http://xena.ucsc.edu) browser to probe RNAseq (PolyA + IlluminaHiSeq Percentile) data.

### 2.5 Statistical analyses

Associations between clinicopathologic characteristics and STING or cGAS expression were analyzed using the *χ* ^2^ test. Receiver operating characteristic (ROC) curves were used to define optimal survival cutoffs for STING mRNA expressions, and survival analysis was performed using the Kaplan–Meier method and the log‐rank test. OS was calculated from the date of diagnosis to the date of death or last available follow‐up. Hazard ratios and confidence interval (CI) were calculated using Cox's proportional hazards method. A two‐sided *p* value of 0.05 was statistically significant. We used SPSS, version 24.0.0.0 (IBM Corporation).

## 3 RESULTS

### 3.1 TCGA analysis of STING mRNA expression in NSCLC

**Figure 2:**
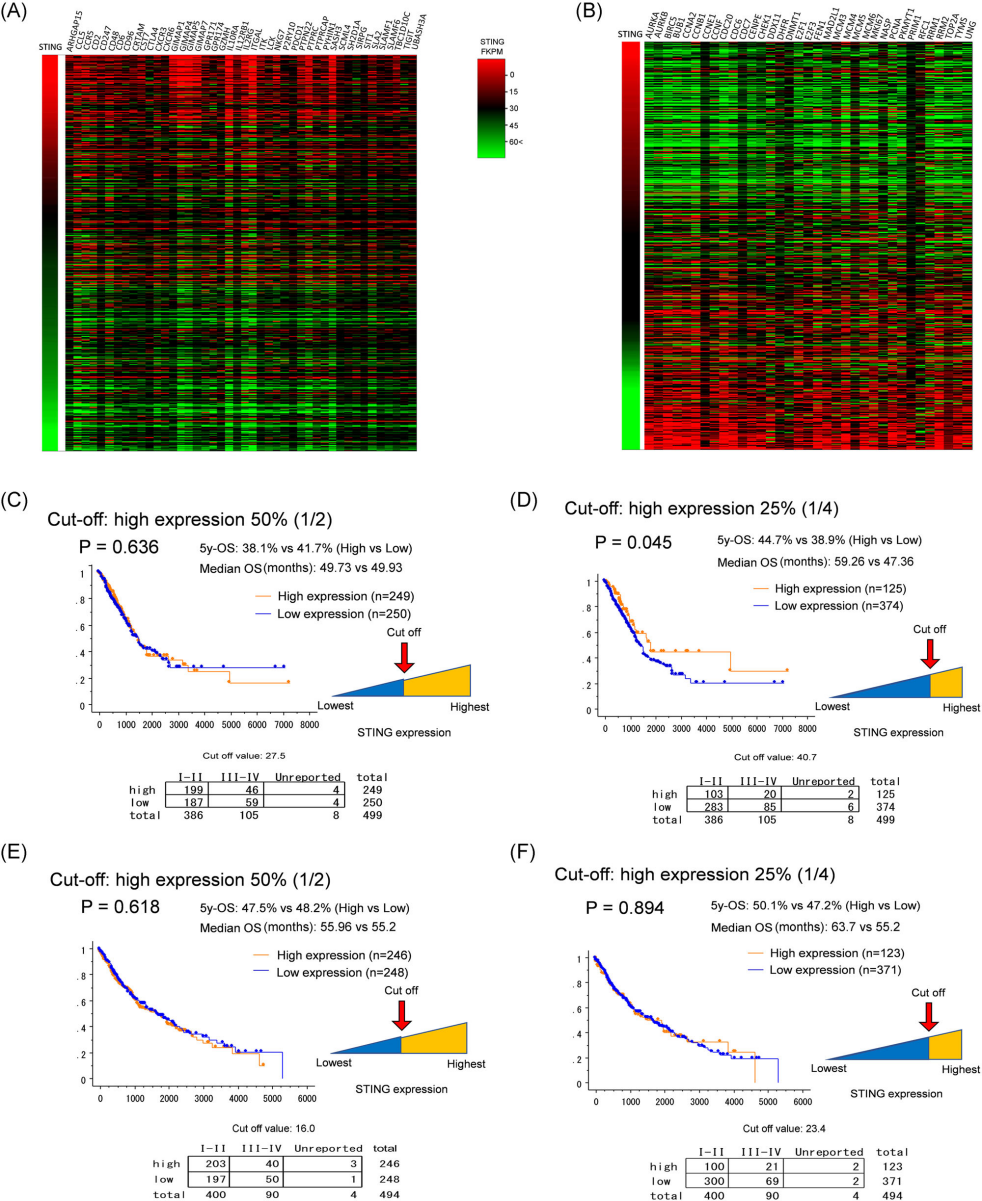
TCGA data on T‐cell signature, cell proliferation genes, and survival according to STING mRNA expression in NSCLC. TCGA RNASeq analysis of STING expression correlates positively with upregulation of genes associated with T‐cell activation in NSCLC (A). High relative STING Expression correlates negatively with common tumor proliferation markers (B). Vertical axis represents patient STING expression scores in FKPM derived from TCGA database. Horizontal axis represents expression of genes associated with T‐cell development and cell proliferation in the same TCGA patient cohort. I AC patients, survival analysis showed no significant difference in 5‐year‐OS between the top 50% and bottom 50% (38.1% vs. 41.7%, *p* = 0.636, C) when stratified by STING RNA expression. However, 5‐year‐OS of the top quartile was significantly improved compared with the bottom 75% (44.7% vs. 38.9%, *p* = 0.045, D). In the same comparisons, high STING‐expressor SCC patients exhibited no significant benefit in 5‐year‐OS neither in the top 50% (47.5% vs. 48.2%, *p* = 0.618, E), nor in the top 25% (50.1% vs. 47.2%, *p* = 0.894, F). According to *χ* ^2^ test, STING‐high vs low was not different in terms of stages in any comparisons. AC, adenocarcinoma; mRNA, messenger RNA; NSCLC, non–small cell lung cancer; OS, overall survival; SCC, squamous cell carcinoma; STING, stimulator of interferon genes; TCGA, The Cancer Genome Atlas

Figure 1 shows experimental design, cohorts, and inclusion criteria. First, we analyzed the effect of STING expression on the immune microenvironment of both AC (*n* = 576) and SCC (*n* = 553). Johnston et al. identified a gene set of lung cancer‐associated T cell signature genes that indicate T cell function in lung tumors.^21^ Analyzing this gene set in terms of STING expression, we see upregulation of most of these genes as STING expression increases, with the highest correlation seen in GTPase Immune‐associated proteins (GIMAPs) important to T‐Cell development, GIMAPs 1,4,5, and 7 (Figures 2A and S1). Other strong correlations with STING expression include interleukin receptors IL10RA, IL2RG, Th1‐biased, pro‐inflammatory chemokine receptor CXCR3, integrin alpha L (ITGAL) involved in cellular adhesion, pan‐leukocyte marker CD45 (PTPRC), and CD96 involved in T‐cell and NK‐cell activation. Thus, the expression of STING shows a positive correlation with T cell function and development. To examine STING's role in tumor growth, we next extended our TCGA analysis to correlations between STING and reported common tumor proliferation markers (Figure 2B). In both AC and SCC (Figure S2), STING expression showed a strong negative correlation with tumor proliferation markers, supporting STING's role as an immune promoter and tumor suppressor.

To evaluate the effect of STING on survival in NSCLC, we analyzed RNA expression data sets available from TCGA (Figure 1A). We clustered TCGA data of AC patients' STING RNA expression according to percentiles, where there was no significant difference in 5‐year‐OS between the top 50% (*n* = 249, *n* = 199 Stage I–II and *n* = 46 Stage III–IV), and bottom 50% (*n* = 250, *n* = 187 Stage I–II and *n* = 59 Stage III–IV), with a cutoff value of 27.5 FKPM (38.1% vs. 41.7%, median OS: 49.73 ± 5.3 vs. 49.93 ± 2.63 months, *p* = 0.636, Figure 2C). In contrast, 5‐year‐OS of the top 25% at a cutoff value: 40.7 FKPM (*n* = 125, *n* = 103 Stage I–II and *n* = 20 Stage III–IV) was significantly higher compared with the bottom 75% (*n* = 374, *n* = 283 Stage I–II and *n* = 85 Stage III–IV) in terms of STING expression (44.7% vs. 38.9%, median OS: 59.26 ± 5.2 vs. 47.36 ± 5.5 months, *p* = 0.045, Figure 2D). TCGA data from patients with SCC revealed no significant difference in 5‐year‐OS above (*n* = 246, *n* = 197 Stage I–II and *n* = 40 Stage III–IV) and below the 50th percentile (*n* = 248, *n* = 197 Stage I–II and *n* = 50 Stage III–IV;) according to STING RNA (median OS: 55.96 ± 12.7 vs. 55.2 ± 4.03 months, *p* = 0.618, Figure 2E). In contrast with AC, there was no significant difference in 5‐year‐OS between the top 25% (*n* = 123, *n* = 100 Stage I–II and *n* = 21 Stage III–IV) and bottom 75% (*n* = 371, *n* = 300 Stage I–II and *n* = 69 Stage III–IV) STING‐expressor patients with SCC (median OS: 63.7 ± 18.2 vs. 55.2 ± 2, *p* = 0.894, Figure 2F). The latter suggests that STING RNA expression is a favorable prognosticator in AC but not in SCC.

### 3.2 IHC expression of STING and cGAS in NSCLC cell lines and tissues

**Figure 3:**
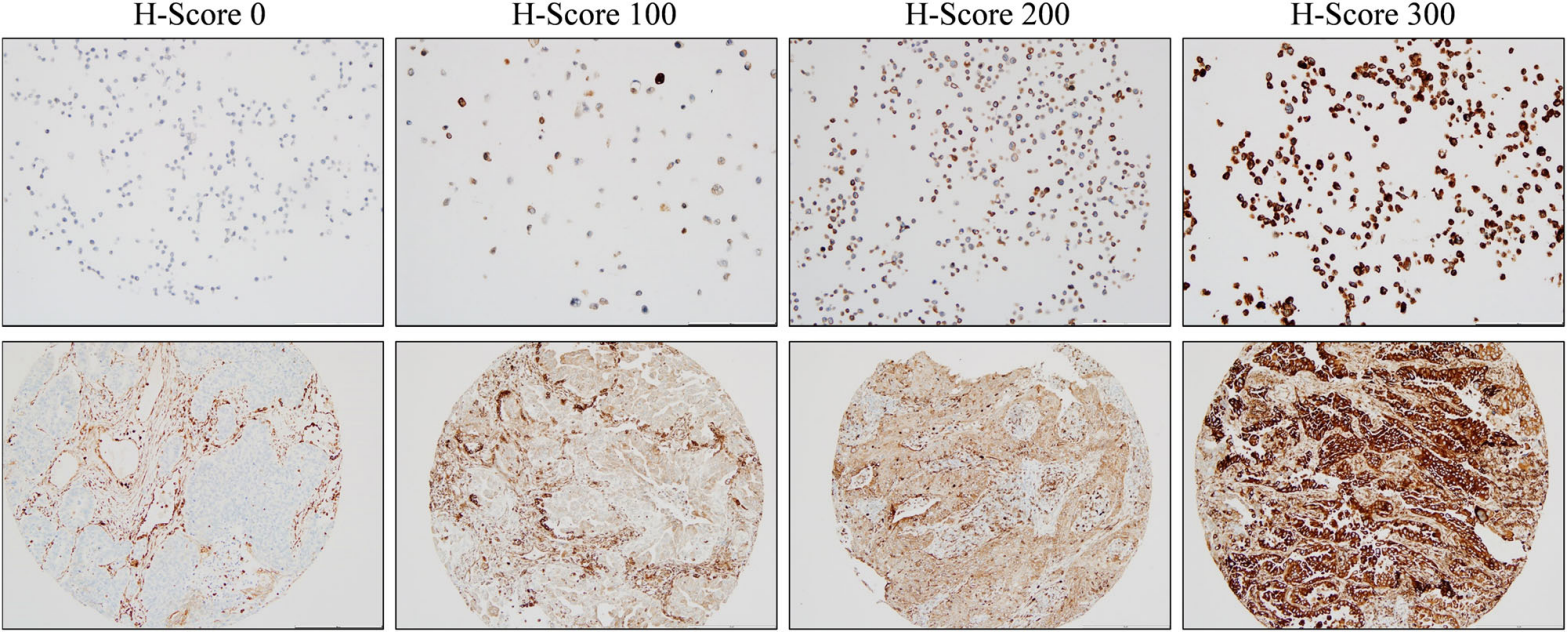
STING IHC shows varying levels of expression in NSCLC cell lines and tissues. Immunohistochemistry for STING expression was performed on TMAs of 55 NSCLC cell lines and 721 individual NSCLC cases. Immune infiltrate staining of STING was widely present, whereas the tumor cells showed a range of STING expression from 0 to 300 H‐Score. In our analysis, patients with H‐score > 50 were considered “STING‐positive.” IHC, immunohistochemistry; NSCLC, non–small cell lung cancer; STING, stimulator of interferon genes; TMAs, tissue microarrays

**Figure 4:**
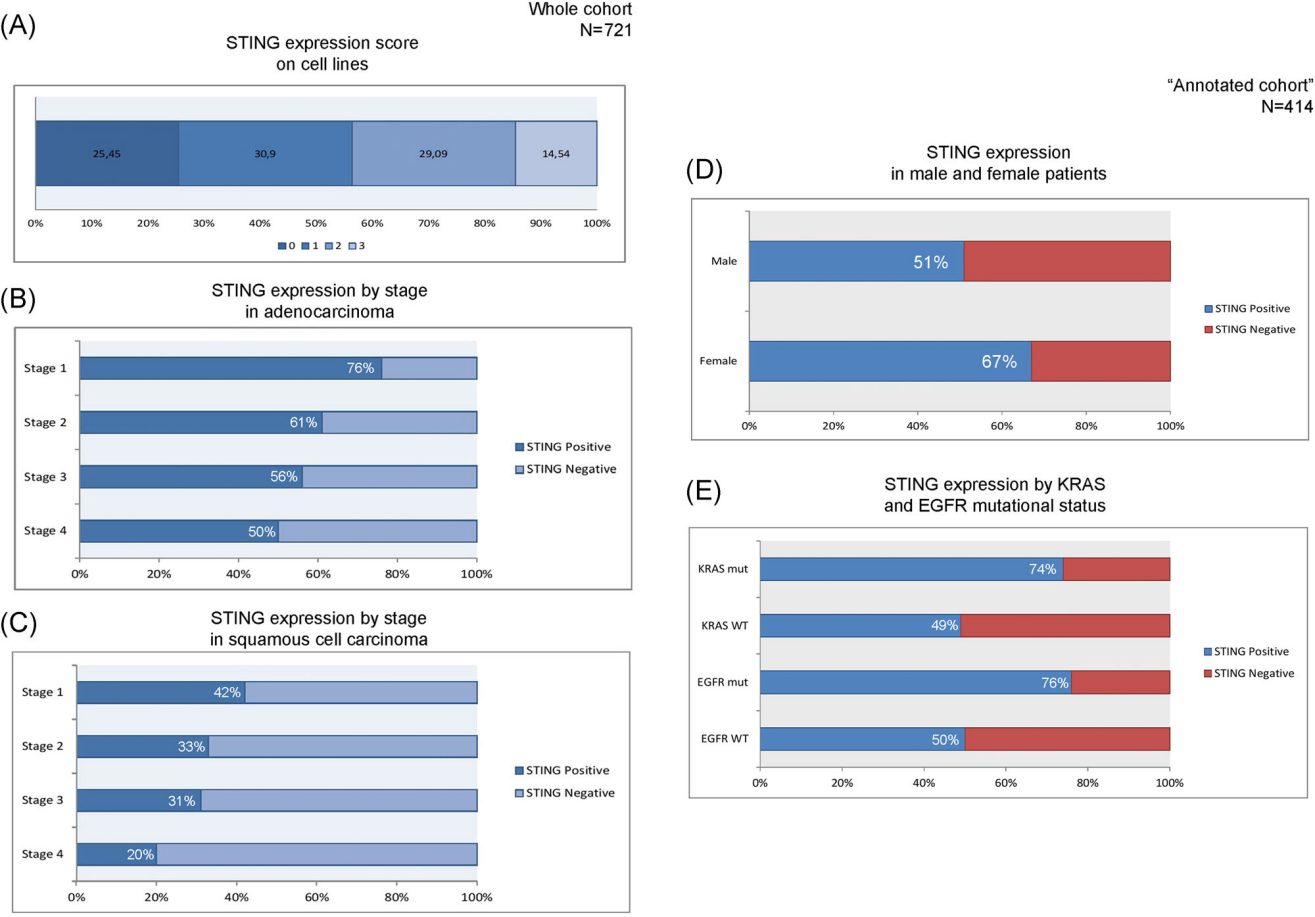
STING Expression by IHC in NSCLC tumor samples. Immunohistochemistry for STING expression was performed on a collection of 55 NSCLC cell lines (A) and 721 individual NSCLC cases with stage and histology data provided. (B) An increased frequency of STING loss in the higher tumor stage occurred in AC and SCC subsets (C). SCC (vs. AC) histology was associated with a significantly lower expression level of STING irrespective of tumor stage. STING expression was lost in an average of 34% of AC tumors compared to 65% loss in SCC histology (D). In the annotated cohort containing TMAs with triplicate cases from 414 NSCLC patients, the STING‐positive case number was significantly higher in females compared to males (66.9% vs. 51.2%, *p* = 0.003) (E) STING positivity was significantly higher in EGFR mutant versus wild type (76.2% vs. 50.0%, *p* = 0.04) and in KRAS mutant versus wild type (74.4% vs. 49.4%, *p* = 0.001) patients. AC, adenocarcinoma; NSCLC, non–small cell lung cancer; SCC, squamous cell carcinoma; STING, stimulator of interferon genes; TMAs, tissue microarrays

We investigated the expression of STING and cGAS using IHC (Figure 3) in 55 NSCLC cell lines and 721 NSCLC tissues (Figure 1B,C). With the H‐score cutoff for positive expression at H‐score = 50, 14/55 (25.45%) of the NSCLC cell lines stained showed negative STING expression (Figure 4A). Evaluation of tissue specimens identified that STING expression in AC shows STING loss as tumor stage increases (positive: 76% Stage I, 61% Stage II, 56% Stage III, 50% Stage IV, 66% total; *n* = 419) (Figure 4B). STING expression is lower at all stages in SCC but still decreases by stage (positive: 42% Stage I, 33% Stage II, 31% Stage III, 20% Stage IV, and 35% total; *n* = 302) (Figure 4C). Expression of cGAS was higher in AC (94%) than SCC (75%) and showed no correlation with stage (Figure S3).

### 3.3 Clinical relevance of STING expression in NSCLC tissues

The “annotated cohort” of 417 cases was analyzed to correlate STING expression with detailed clinicopathological characteristics and clinical outcomes. The mean STING H‐score in this cohort was 104.3 with a standard deviation of 103.1. In this cohort, statistically relevant correlations were found between STING and multiple clinical data points. The proportion of cases with STING positivity was higher in females compared to males (66.9% vs. 51.2%, *p* = 0.003) (Figure 4D), and in nonsquamous versus squamous histologies (62.0% vs. 44.7%, *p* < 0.001). STING H‐score in this cohort was much higher in early‐stage cases and decreased with more advanced stage (positive in 64.3% Stage I, 52.9% Stage II, 45.6% Stage III, 66.6% and Stage IV, *p* = 0.005) (Tables S1–S2). Both AC and SCC subsets showed increasing STING loss with advanced stage, with AC showing higher STING expression at all stages than SCC. The proportion of cases with STING positivity was significantly higher in EGFR mutant versus wild type (76.2% vs. 50.0%, *p* = 0.04) and in KRAS mutant versus wild type (74.4% vs. 49.4%, *p* = 0.001) (Figure 4E).

**Figure 5:**
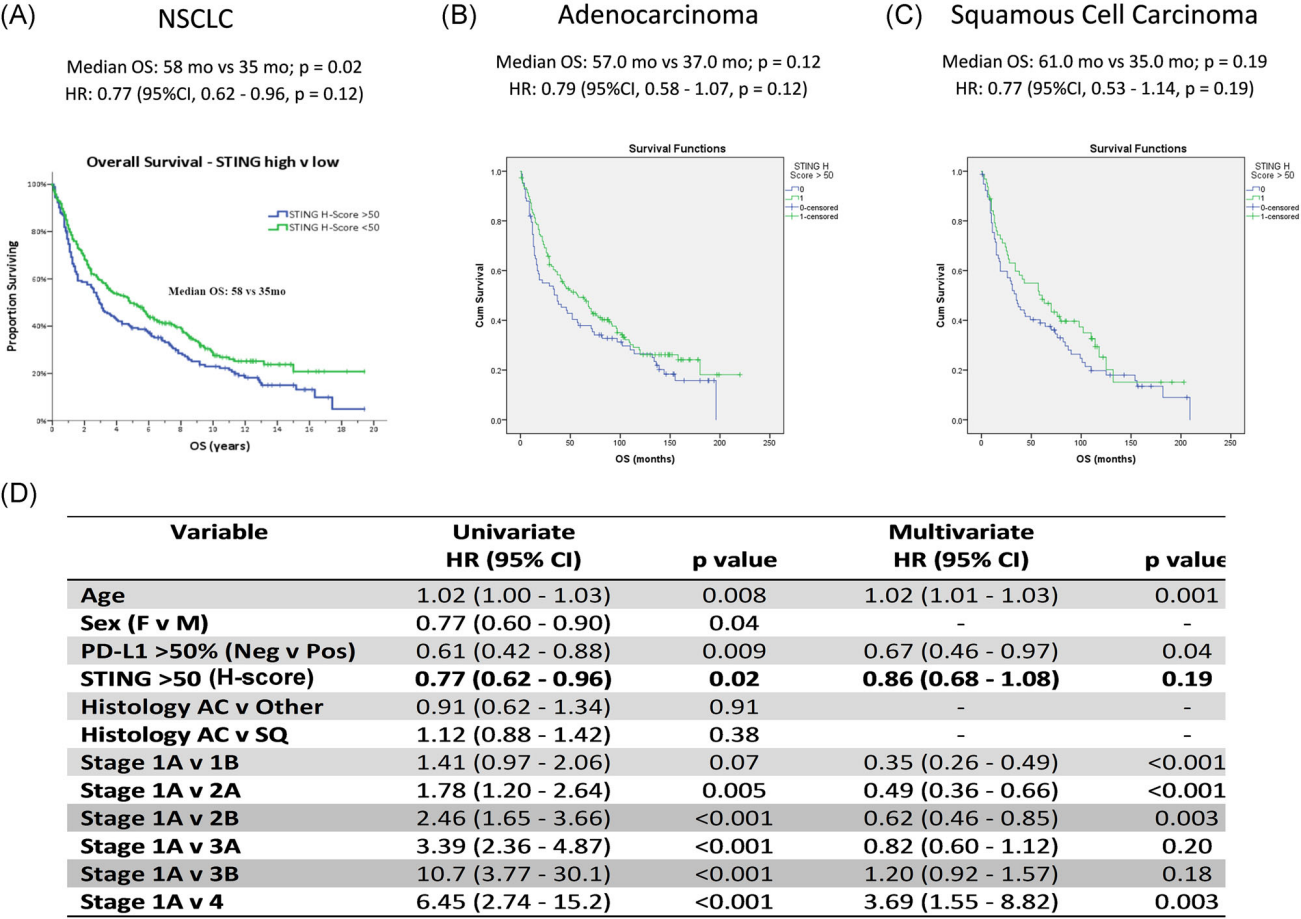
Expression of STING protein is prognostic in NSCLC. A total of 421 NSCLC patients (“annotated” cohort) were analyzed for STING expression, where positive STING expression (H‐score > 50) conferred significantly increased OS (58 months vs. 35 months, *p* = 0.02) for patients (A). In both histological subsets (AC and SCC) a nonsignificant survival benefit was seen in those patients expressing STING over the cutoff of 50 H‐score: AC: median OS: 57.0 months versus 37.0 months; *p* = 0.12 HR: 0.79 (95% CI, 0.58–1.07, *p* = 0.12, B); SCC: median OS: 61.0 months versus 35.0 months; *p* = 0.19 HR: 0.77 (95% CI, 0.53–1.14, *p* = 0.19, C). Uni‐ and multivariate analysis concerning main clinicopathological parameters and STING expression is shown in panel (D). AC, adenocarcinoma; CI, confidence interval; HR, hazard ratio; NSCLC, non–small cell lung cancer; SCC, squamous cell carcinoma; STING, stimulator of interferon genes

STING‐positive patients (H‐score > 50) showed significantly increased survival in the “annotated” cohort (*n* = 421) (median OS: 58 vs. 35 months, *p* = 0.02) (Figure 5A). When patients were stratified by histology, STING positive patients with both AC (median OS: 57 vs. 37 months, *p* = 0.12, Figure 5B) and SCC (median OS: 61 vs. 35 months, *p* = 0.19 Figure 5C) showed a nonsignificant increase in OS. Figure 5D shows survival relative to clinicopathological characteristics according to univariate and multivariate analysis. STING‐ high expression was prognostic in the univariate analysis and we found conventionally strong prognosticators such as stage, age, and PD‐L1 significant in the multivariate analysis too.

### 3.4 Methylation of STING and cGAS promoters in NSCLC

**Figure 6:**
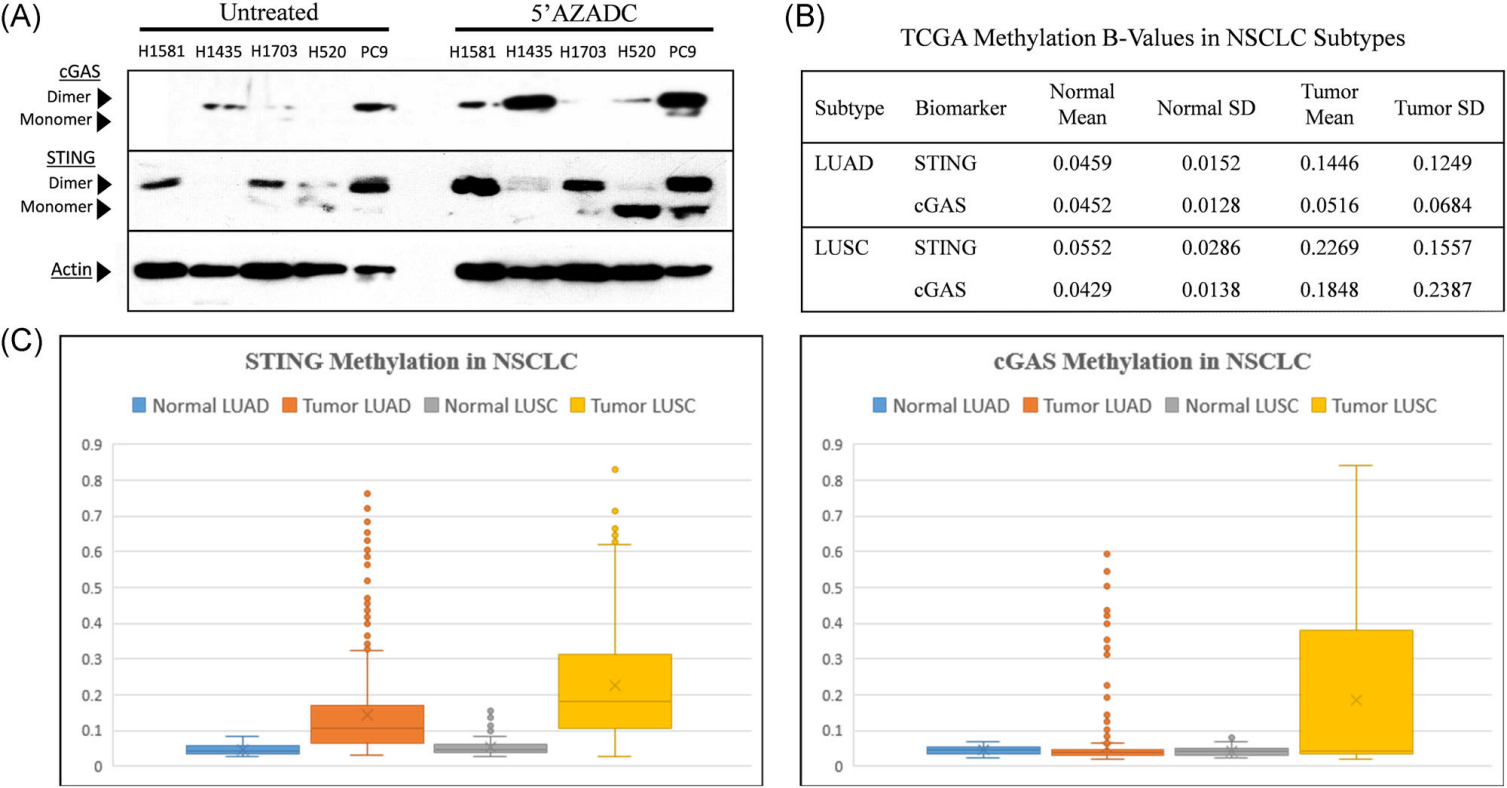
Restoration of STING and cGAS expression by demethylation (A) Selected cell lines were either incubated in standard media or media containing 10 µM of demethylating agent 5ʹAZADC. 40 µg cell lysate was analyzed for cGAS and STING expression via western blot. Restoration of STING and cGAS was seen in the majority of cell lines treated. (B) and (C) TCGA Illumina 450k Methylation database was examined for methylation of STING and cGAS promoters in both adenocarcinoma (LUAD) and squamous cell carcinoma (LUSC) data sets. Methylation of promoters was much higher in tumors than normal, with SCC showing higher average methylation than AC. In AC, STING methylation was much more common than cGAS. AC, adenocarcinoma; cGAS, cyclic GMP‐AMP synthase; NSCLC, non–small cell lung cancer; STING, stimulator of interferon genes; TCGA, The Cancer Genome Atlas

Since methylation of STING and cGAS promoters has been implicated in the loss of STING or cGAS protein expression, we analyzed TCGA NSCLC methylation data and sought to restore STING or cGAS expression in cell lines using the demethylating agent 5ʹAZADC (Figure 6). TCGA analysis of the Illumina Methylation 450k database shows STING promoter hypermethylation in AC (0.15 ± 0.13 tumor vs. 0.05 ± 0.02 normal, *n* = 422) and SCC (0.23 ± 0.16 tumor vs. 0.04 ± 0.03 normal, *n* = 359). The cGAS promoter shows slight methylation in AC (0.05 ± 0.07 tumor vs. 0.05 ± 0.01 normal, *n* = 422) but a large increase in SCC (0.19 ± 0.24 tumor vs. 0.04 ± 0.01 normal, *n* = 359). Here we investigated methylation of STING and cGAS in five STING‐negative NSCLC cell lines confirmed by IHC. Cell lines were treated with demethylating agent 5ʹAZADC followed by western blot analysis of STING and cGAS protein expression. STING expression increased from baseline in each of the 5ʹAZADC‐treated cell lines, while cGAS expression increased in 4/5 of the cell lines.

## 4 DISCUSSION

T cell responses and the immune microenvironment are critical factors for therapeutic efficacy. The reported response rates for anti‐PD immunotherapy in NSCLC are still about 20%.^22^ Altering immune response might help in understanding the underlying mechanism for tumor progression. While analysis of STING expression and correlation with clinical outcomes have been published in some small cohorts of other cancers, we have examined STING expression in a large NSCLC patient data set of *n* = 721 individual cases. This is complemented with TCGA analysis and cell line functional data to validate our results. We found that the STING is extensively expressed in NSCLC, and the proportion of STING‐positive cases was more significant in AC than SCC in both individual cohorts. In addition, we showed that STING expression is reduced with a higher tumor stage, irrespective of histology. These data may have future relevance with the emergence of STING agonist therapy and or in a potential combination with anti‐PD immunotherapy.

The STING pathway might be defective in lung tumors that can alter responses, as reported in other cancers. Others showed that STING/cGAS expression was lost in tumor tissues; however many of these studies involve only a small number of cases.^23^, ^24^, ^25^ To our knowledge, STING expression has not been extensively studied in larger patient cohorts with clinicopathological data correlation. TCGA data analysis of STING and cGAS in AC and SCC shows that low expression of STING in adenocarcinoma, but not squamous cell carcinoma, correlates with poor survival. Further TCGA analysis shows STING expression correlates positively with expression genes identified as “T‐cell signature genes” in lung cancer, while STING expression negatively correlates with common tumor proliferation genes.^26^ Analysis of TCGA Illumina Methylation 450k database shows increased methylation of STING and cGAS genes in AC and SCC.

Activating STING to transform immunologically refractive cold tumors to a hot phenotype is a potential new therapeutic approach.^27^, ^28^ Inhibitors to the enzyme poly‐ADP ribose polymerase (PARP) are new agents proved experimentally to reduce DNA damage repair, potentially increasing STING activation.^29^ Another possibility is the inhibition of DNA damage response (DDR) proteins that were shown to activate the STING/TBK1/IRF3 molecular pathway, followed by a significant increase in chemokine levels (CCL5 and CXCL10), and cytotoxic T‐cell activation. Experiments using cell lines and in vivo mouse models also demonstrated that knockdown of STING and cGAS reversed the tumoricidal effect of DDR and PD‐L1 blockade.^30^ Moreover, STING‐deficient mice are more susceptible to tumor formation, exhibiting a decreased T‐cell‐mediated antitumor immunity and impaired responses to immunotherapy.^31^

Positive STING protein expression (H‐score > 50) was associated with significantly improved overall survival (58 vs. 35 months, *p* = 0.02) in a pooled NSCLC cohort, and a nonsignificant improve in overall survival when stratified according to histologies (AC: 57 vs. 37 months; SCC: 61 vs. 35 months). We found stage, age. and PD‐L1 significant in the multivariate analysis, but STING‐high expression in NSCLC remained prognostic only in the univariate analysis, possibly due to lack of STING specific treatment. Analysis of TCGA RNA expression data of *n* = 499 AC patients confirmed our finding that patients in the upper quartile (STING expression) exhibit significantly better 5‐year‐OS, including early‐ and late‐stage diseases. In contrast, STING RNA expression was not proved to be a positive prognosticator in SCC in none of the comparisons according to the same analysis. Furthermore, TCGA RNA expression data showed that STING was associated with T‐cell promotion and development genes underpinning its rule as an immune activator. Since STING is a consequence of detecting non‐self DNA in the tumor microenvironment, STING expression could be increased in those tumors with common mutations, such as TP53.^25^ In our study, STING expression correlated significantly with the presence of EGFR or KRAS mutations that is in contrast with the results of other groups, claiming that KRAS and STK11 mutations, with LKB1‐loss, are enriched in STING‐low tumors.^23^, ^25^, ^32^ This might be explained by the difference between tumor types and case numbers, and our annotated cohort represented more early stage NSCLC patients, where STING‐expression might be higher and independent of tumor mutational status.

The underlying mechanism for STING and cGAS loss has been attributed to hypermethylation of the promoter regions for both genes. Given that epigenetic modifications are common in NSCLC, it is reasonable to expect methylation to play a role in STING and cGAS expression. TCGA analysis of methylation for both STING and cGAS genes showed much higher methylation in SCC than in AC. The differences in methylation between AC and SCC might explain why STING expression is more remarkable in AC than in SCC. Recent studies combining PD‐L1 inhibitors with low‐dose demethylating agents such as azacytadine have been shown to improve outcomes.^33^ Combinations of STING agonists with low‐dose Azacytadine could increase STING activity and sensitize low STING expressers, especially SCC tumors, to STING‐targeted therapies.

Moreover, a recent study showed that cisplatin treatment increases the activation of the STING/cGAS pathway and is associated with higher PD‐L1 expression in multiple NSCLC preclinical models in both AC and SCC.^25^ Small molecule substances, such as cyclic dinucleotides (CDNs) derived from bacteria might directly activate the STING signaling pathway. CDNs increased the infiltration of tumor‐specific cytotoxic T‐cells and enhanced the therapeutic efficacy of anti‐PD‐1 and anti‐CTLA‐4, reprogramming immunosuppressive, M2‐polarized tumor‐associated macrophages to a pro‐inflammatory M1‐macrophages.^34^ It was also reported that STING‐activating nano‐vaccines delivering tumor neo‐antigens could effectuate intense and persistent antigen‐specific T‐cell responses, which were followed by vigorous immunotherapeutic efficacy in numerous murine cancer models.^35^, ^36^

Of note, our study has possible clinical and therapeutic implications. Others showed in a preclinical ovarian cancer study that survival of mice treated with a combination of STING, carboplatin, agonist, and anti‐PD‐1 antibody was the longest.^37^ In the clinical setting, a number of effective small‐molecule STING agonists emerged, already under phase I‐III clinical trials, including ADU‐S100 (NCT03937141), BMS‐986301 (NCT03956680), and DMXAA specifically trialed in NSCLC (NCT00674102) combined with carboplatin and paclitaxel.^38^ CDN‐based STING agonist, MK‐1454 combined with pembrolizumab is progressing from Phase I to Phase II clinical trial (NCT04220866) in advanced solid tumor indication.^39^ However, systemic administration of STING agonists may induce pathological inflammation. This concern is based on the fact that overactivation of STING might be involved in a broad range of autoimmune conditions.^40^ Still, a safe, effective, and efficient way of STING‐agonism in advanced‐stage solid tumors, including NSCLC could strongly potentiate PD‐L1 immunotherapies and transform these aggressive malignancies into chronic conditions.

Limitations of this study include that we have no comprehensive data collected on specific treatments in subgroups. Moreover, our "annotated cohort" is from overwhelmingly early‐stage NSCLC patients, and we have no data on the potential clinical behavior without STING targeted specific treatments administered in advanced‐stage patients.

## 5 CONCLUSIONS

Our sizable clinical patient cohort shows that STING is extensively expressed in NSCLC. High STING tumor expression correlates with improved survival, early‐stage disease, and EGFR and KRAS mutations. Our data are further supported by the coincidence of STING and T‐cell activation genes, cell line demethylation, and TCGA data. Furthermore, our study provides multiple aspects of STING expression's translational relevance. Along with other studies on immunotherapy and STING associations, our data serve as a reasonable basis for further exploring the exact clinical role of STING in NSCLC.

## CONFLICT OF INTERESTS

The authors declare no conflict of interest.

## AUTHOR CONTRIBUTIONS

*Conceptualization*: David Dora, Christopher J. Rivard, Fred R. Hirsch, and Zoltan Lohinai. *Methodology*: Charles Caldwell jr, Christopher J. Rivard, Kenichi Suda, and Hui Yu. *Validation*: Charles Caldwell jr, Christopher J. Rivard, Gareth Rivalland, and Kim Ellison. *Formal analysis*: Hui Yu, Gareth Rivalland, Leslie Rozeboom, Rafal Dziadziuszko, and Paul Mitchell. *Investigation*: Charles Caldwell jr, Christopher J. Rivard, Zoltan Lohinai, and David Dora. *Resources*: Christopher J. Rivard and Fred R. Hirsch. *Data curation*: Hui Yu, Gareth Rivalland, Paul Mitchell, Thomas John, Inigo San Millan, and Shengxiang Ren. *Visualization*: Charles Caldwell jr, Christopher J. Rivard, and David Dora. *Supervision*: Zoltan Lohinai, Christopher J. Rivard, David Dora, and Fred R. Hirsch. *Project administration*: David Dora, Zoltan Lohinai, Christopher J. Rivard, Leslie Rozeboom, Rafal Dziadziuszko, Paul Mitchell, Thomas John, Inigo San Millan, and Shengxiang Ren. *Writing‐original draft*: Charles Caldwell jr, David Dora, Zoltan Lohinai, and Christopher J. Rivard. *Writing‐review and editing*: David Dora, Zoltan Lohinai, and Christopher J. Rivard.

## SYNOPSIS

This study evaluates RNA and tissue protein expression of the immune‐checkpoint STING in a sizable cohort of NSCLC patients. We find that increased STING expression is associated with increased expression of T‐cell activation genes, early stage, EGFR, and KRAS mutations and improved survival.

## Supporting information

Supporting information.

## Data Availability

Data are available from the corresponding author upon reasonable request.
