# Supplementary material for: Loss of STING expression is prognostic in non–small cell lung cancer
Source: J Surg Oncol. 2022 Jan 31;125(6):1042–52. doi: 10.1002/jso.26804 (PMC9304565; doi:10.1002/jso.26804)
Supplement: Supplementary file 1 — Supporting information. [file JSO-125-1042-s001.docx]

**SUPPORTING MATERIAL**

**
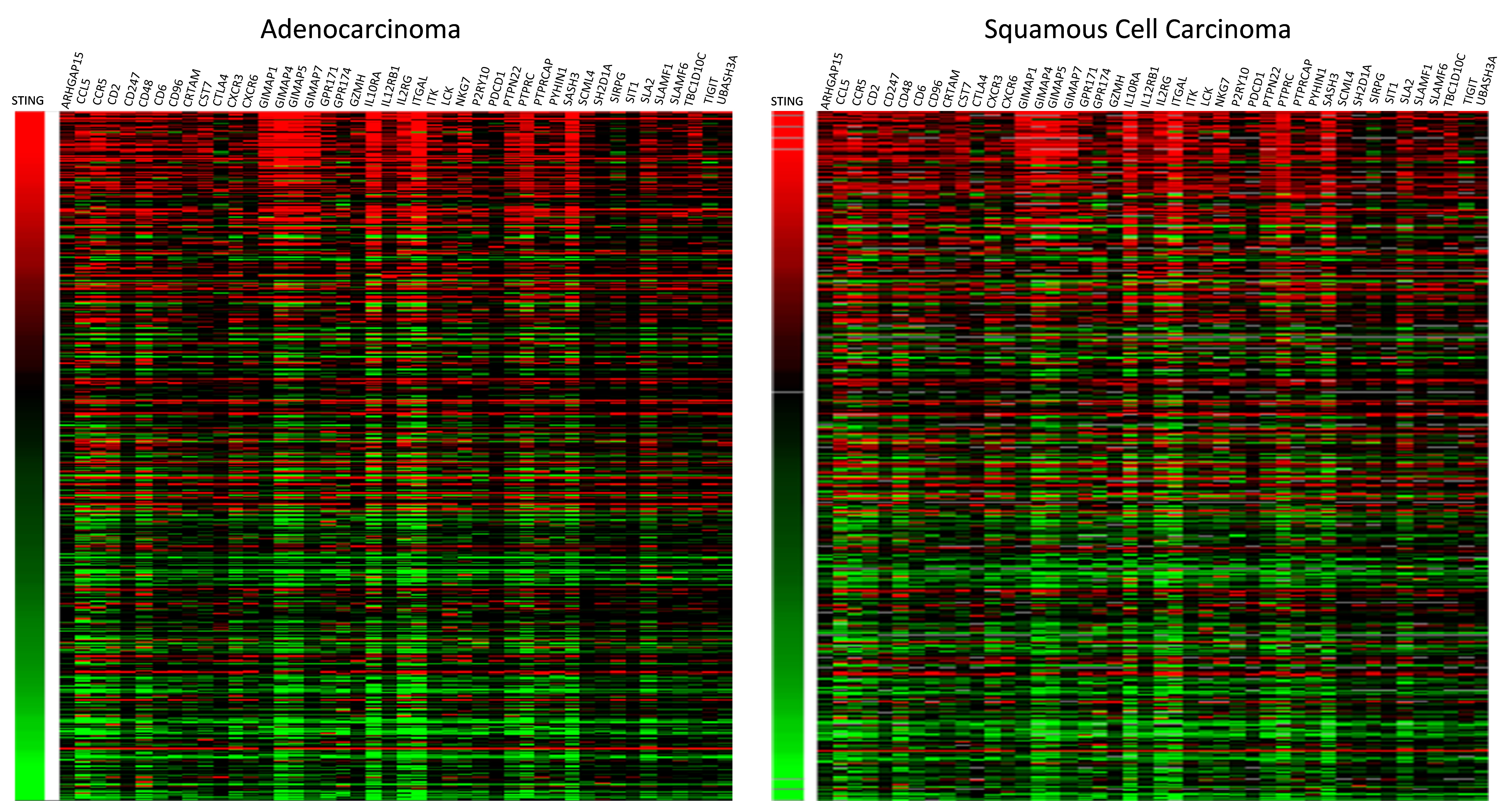
**

**Supporting Fig. 1: NSCLC T-Cell Signature Genes on STING mRNA in TCGA.** STING expression correlates positively with genes identified to be NSCLC T-Cell Signature Genes in both adenocarcinoma and squamous cell carcinoma subsets.


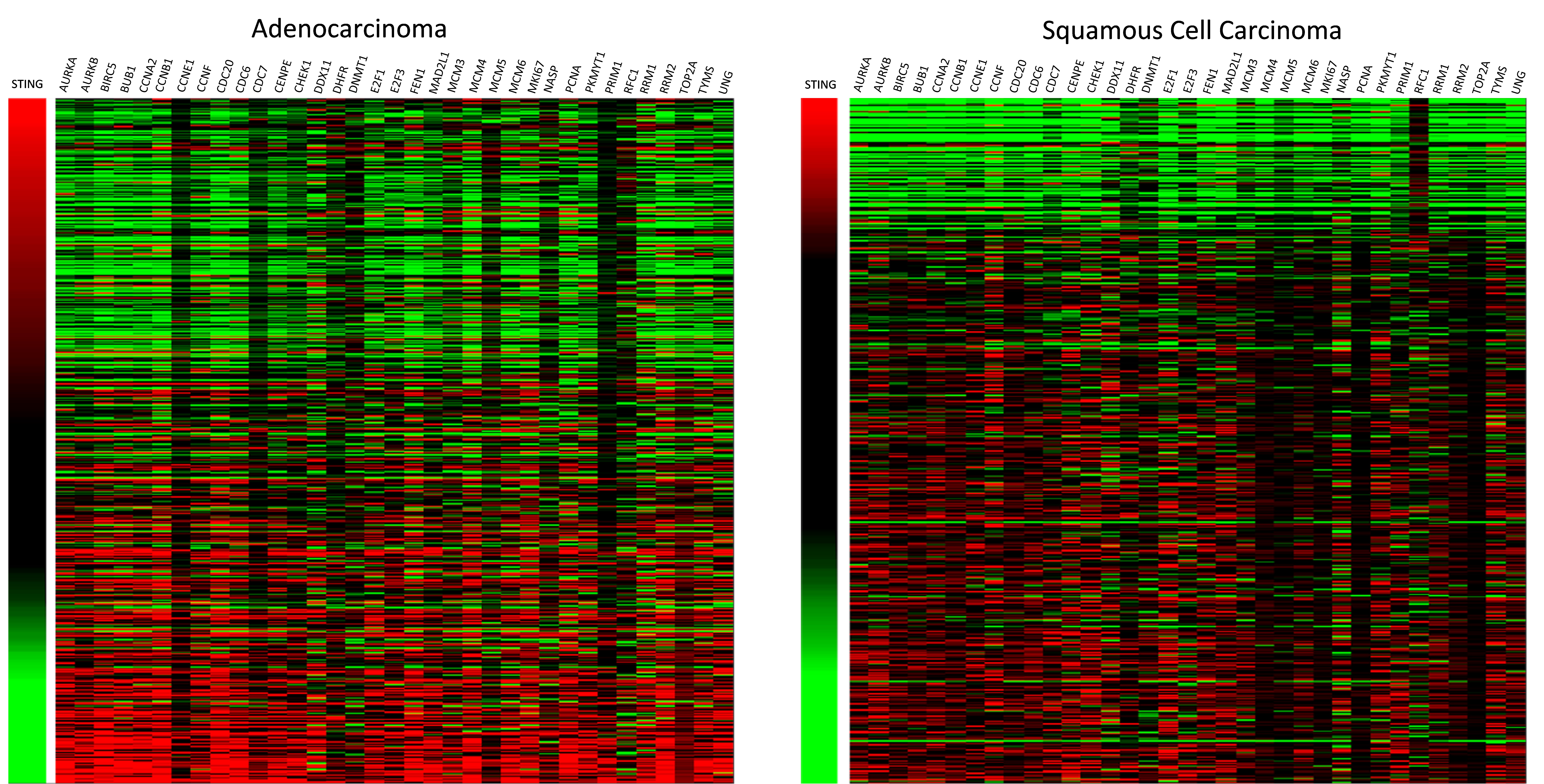


**Supporting Fig. 2: TCGA Tumor Proliferation Genes Based on STING mRNA in NSCLC.** STING expression correlates negatively with common tumor proliferation markers in both adenocarcinoma and squamous cell carcinoma subsets.

**
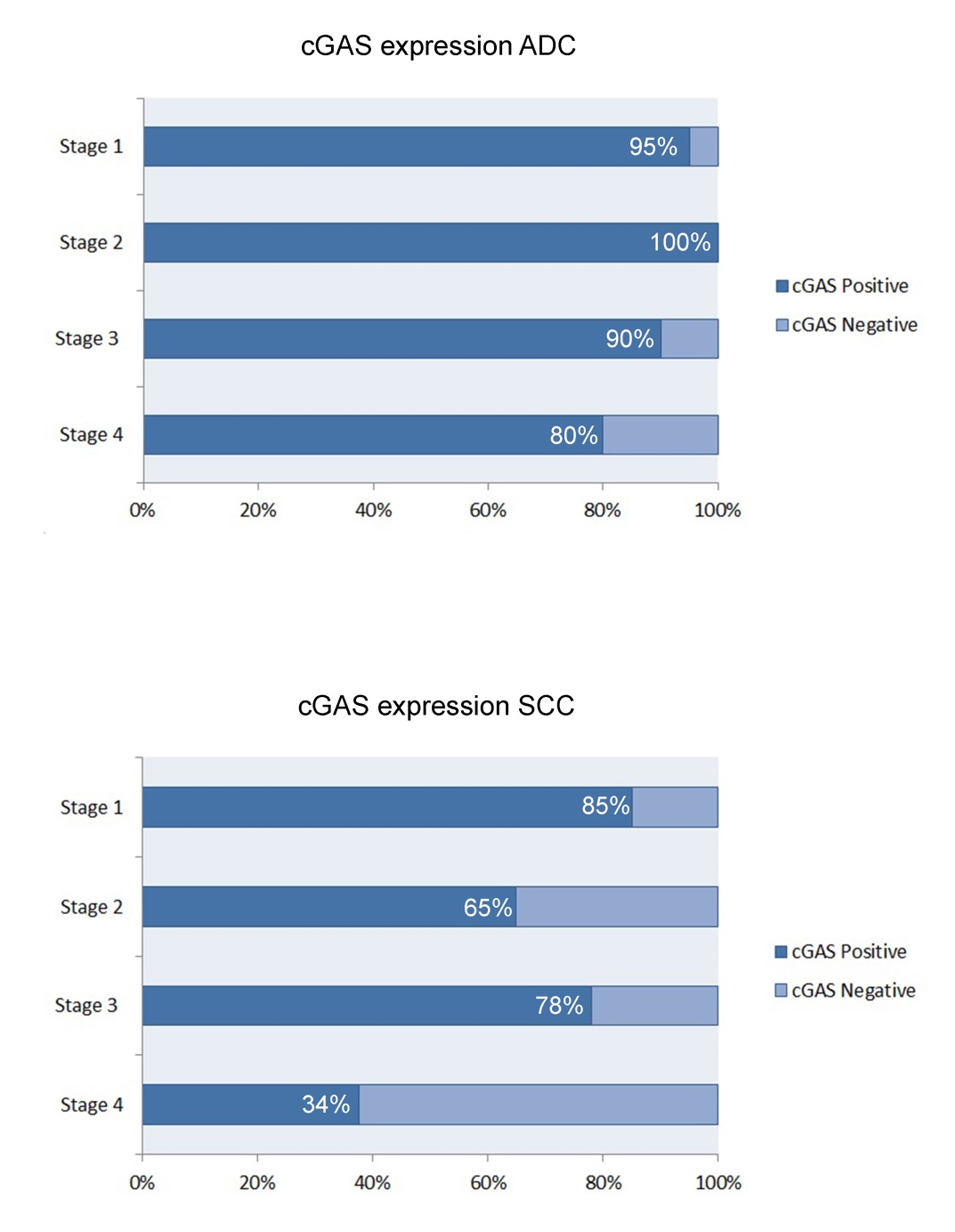
**

**Supporting Fig. 3: cGAS Expression in NSCLC clinical cohort.** A cohort of 721 patients was analyzed for cGAS expression by IHC. cGAS was positive in the majority of ADC tumors regardless of stage. However, patients with SCC show decreased cGAS expression compared to ADC and a lower number of cGAS-positive cases in stage 4.


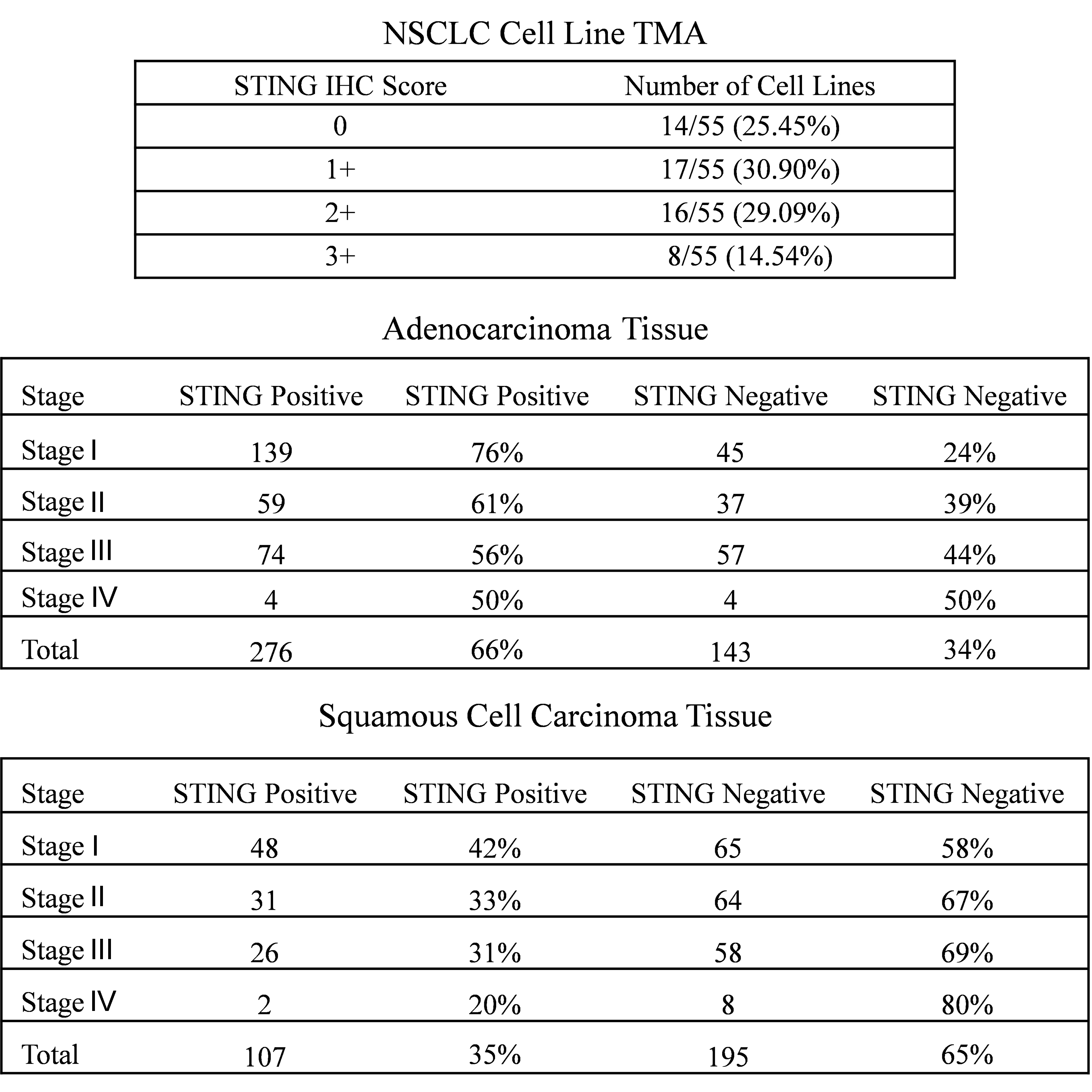


**Supporting Table 1: STING protein expression in cell lines and in the total cohort according to stage and histology.** 0, 1+, 2+ and 3+ STING expression scores on cell lines correspond to 0, 100+, 200+ and 300+ H-scores on tissue samples. The table shows quantitative data of all 721 tissue samples, including the "tumor" and the "annotated cohorts". A cutoff of H-score > 50 defines STING-positivity.


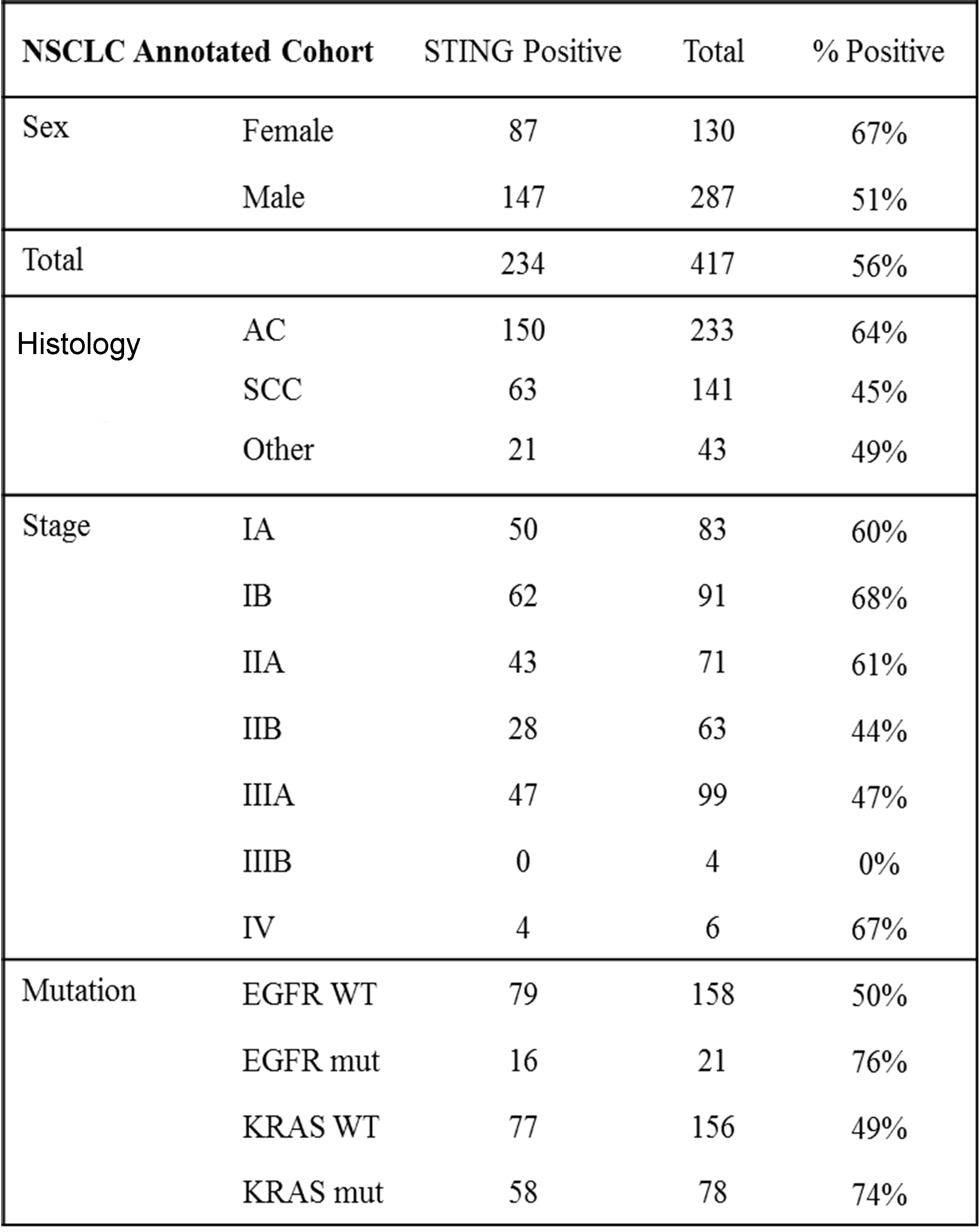


**Supporting Table 2: STING positive cases in the cohort with available clinicopathological data (‘annotated cohort’) according to the stage, sex, histology, and mutation type.** A cutoff of H-score > 50 defines STING-positivity.
